# Exploring the weight bias of professionals working in the field of obesity with a mobile IAT: a pilot study

**DOI:** 10.1177/20420188221098881

**Published:** 2022-05-14

**Authors:** Tobias Jungnickel, Ute von Jan, Stefan Engeli, Urs-Vito Albrecht

**Affiliations:** Peter L. Reichertz Institute for Medical Informatics of the TU Braunschweig and Hannover Medical School, Hannover Medical School, Hannover, Germany; Peter L. Reichertz Institute for Medical Informatics of the TU Braunschweig and Hannover Medical School, Hannover Medical School, Hannover, Germany; Institute of Pharmacology, Center of Drug Absorption and Transport (C DAT), University Medicine Greifswald, Felix-Hausdorff-Str. 3, 17487 Greifswald, Germany; Peter L. Reichertz Institute for Medical Informatics of the TU Braunschweig and Hannover Medical School, Hannover Medical School, Hannover, Germany; Department of Digital Medicine, Medical Faculty OWL, Bielefeld University, Bielefeld, Germany

**Keywords:** weight bias, implicit bias, Implicit Association Test, ResearchKit, obesity

## Abstract

**Background:**

Obesity is common in many industrialized nations and often accompanied by related health issues. Furthermore, individuals living with overweight or obesity are often confronted with stigmatization in their daily lives. These problems may be aggravated if the objectivity of health care professionals is compromised due to (unconscious) prejudices. If pharmaceutical companies, regulatory agencies, and health insurers are also susceptible to these biases, decisions related to the development, approval, and reimbursement of obesity-related therapies may be negatively impacted.

**Materials and Methods:**

The ‘Implicit Association Test’ (IAT) is a psychometric test allowing to measure these attitudes and could therefore assist to reveal unconscious preferences. A self-developed mobile version, in the form of a ResearchKit-based IAT app was employed in the presented study. The objective was to determine (potential) weight bias and its characteristics for professionals attending a national obesity-related conference in Germany (G1), compared to a control group (without stated interest in the topic, G2) – both using the mobile app – and a historical control (G3) based on data provided by Project Implicit acquired by a web app.

**Results:**

Explicit evaluations of G1 were neutral at a higher percentage compared with G2 and G3, while implicit preference toward lean individuals did not differ significantly between G2 and G3, and G1.

**Conclusion:**

The greater discrepancy between the (more neutral) explicit attitude and the unconscious preference pointing in the anti-obesity direction could indicate an underestimated bias for the professional participants in G1. Implicit preference is often ingrained from childhood on, and difficult to overcome. Thus, even for professionals, it may unconsciously influence decisions made in the care they provide. Professionals in any given health care sector directed at obesity care should thus be made aware of this inconsistency to enable them to consciously counteract this potential effect.

## Introduction

According to the World Health Organization (WHO), ‘overweight and obesity are defined as abnormal or excessive fat accumulation that may impair health’.^1^ Overweight and obesity are endemic in many industrialized nations. Based on the body mass index (BMI), comparable with other EU member states, more than half the adult population appeared to be overweight (35.2%, BMI ⩾ 25 kg/m^2^) or obese (16.9%, BMI ⩾ 30 kg/m^2^) in Germany in 2014,^2^ although, on its own, the BMI should only be used as a rough guide for such classifications: Despite various health problems being associated with obesity, such as, cardiovascular diseases or diabetes mellitus type 2,^3^ not all individuals with a high BMI are actually suffering from obesity. The BMI may, for example, misclassify individuals^4^ with an exceptionally high muscle mass and low fat accumulation, such as professional athletes.^5^ However, while a high BMI may not necessarily have a negative impact on health for these individuals, it may, for example, hinder an individual’s ability to either obtain health insurance (mandatory or optional) at all or to do so at a reasonable premium, depending on what is allowed in the respective jurisdiction.^6^

Nevertheless, for individuals who are actually affected by overweight or obesity, weight-related stigmatization may have even more serious consequences. Open or unwitting stigmatization in society, in the work environment, within the circle of family and friends,^7^ or even in health care, can nourish or further exacerbate existing health problems: Weight-related health and mobility problems, in combination with experienced stigmatization and discrimination in social interactions, may contribute to anxiety and depression. This, in turn, may negatively affect eating behavior and physical activity levels^8^ and may even cause persons with overweight or obesity to delay or avoid much needed healthcare.^9^ Taken together, the aforementioned factors, among others, may further increase weight-related problems in both the physical and psychological domain, and, ultimately, this vicious cycle will often be difficult to break for affected individuals.^10,11^

A negative attitude toward individuals with high body weight is common in many societies, and the corresponding stigmatization has not declined in recent years,^11,12^ although there is also evidence supporting the idea that an increasing exposure to individuals with obesity may lead to an increased acceptance.^13^ In an evaluation of data obtained between 2007 and 2016, Charlesworth and Banaji nevertheless found indications that over time, implicit bias related to body weight – and thus corresponding subconscious prejudice – actually increased in society, that is, resulting in a stronger implicit preference of lean individuals *versus* having a more neutral attitude.^14^

In healthcare contexts, open, explicit bias and unconscious, implicit bias and the ensuing inadvertent stigmatization may be harmful, as they may lead to discrimination against individuals with obesity by health care professionals.^15,16^ This, in turn, may severely impact the quality of care provided to those individuals affected by overweight and obesity.^17,18^ Therefore, analyzing whether there is a weight bias in professionals working in the field of obesity and to determine which preferences prevail are of scientific importance.

As a computerized method of choice for identifying (subconscious) prejudice and a tendency toward stigmatization in specific contexts, the ‘Implicit Association Test’ (IAT), as introduced by Greenwald *et al.*^19,20^ in the late 1990s is popular.^21,22^ The existence and extent of such hidden prejudice does not necessarily correspond to self-reported, openly expressed attitudes (or ‘explicit bias’). On the contrary, these explicitly voiced attitudes are deemed to be more in line with what is socially acceptable or desired rather than an individual’s actual inclination toward or against individuals with certain characteristics.^23^

As originally constructed, the IAT is meant to determine whether, for an individual, there are implicit, subconscious associations between representations of certain concepts and corresponding stereotypes. Such an unreflected attribution of certain characteristics or prejudices to someone (or a group of individuals) for whom certain characteristics are present is also called an ‘implicit bias’.^23^ Using the IAT, it is may thus not only be possible to determine whether such implicit bias exists but the strength of this bias can be tested as well.

Over the years, there have nevertheless been voices that suspect there to be different mechanisms at work. For example, within the literature, Schimmack^24^ has identified at least four interpretations as to what the IAT is actually able to measure: First, Payne *et al.*^25^ question the stability of its results and propose implicit bias to be influenced by situational aspects (e.g. one’s environment) rather than a individual’s (fixed) bias. Second, it is a possibility that the construct itself may be valid, but that the IAT itself is nevertheless a poor measure of it.^26,27^ On a more positive note, there are also those who interpret the IAT to be a valid measure of bias that can just as well be determined using explicit measures [Granados_Samayoa2017-mw], and finally, the initial assumption that it is able to measure implicit bias where explicit measures are insufficiently able to capture bias also remains a possibility.^19^ Schimmack suspects that the IAT’s validity may also depend on the subject area where it is applied.

Despite these open discussions, we decided to use the IAT in our research. It has been widely used in the area of weight-bias-related research, both for the general population and in a professional context.^15,28–36^ Also, its main advantage that it is relatively easy to apply without any specialized equipment makes it attractive for testing in the field: All that is necessary is a device running an implementation of the IAT. Jungnickel *et al.*^37^ developed and evaluated a version for iOS devices for the weight bias topic that allows for more flexibility of IAT applications in the field.

### Objective

We aimed to analyze possible weight bias and its characteristics in a population of professionals working in the field of obesity using the mobile app at a conference (G1), compared to a control group without any specific relationship to the topic – also using the mobile version (G2) – and a historical control group based on data provided by Project Implicit using a web app (G3).

The work presented here is meant to serve as a first evaluation of a mobile, ResearchKit-based implementation of the IAT – as described in Jungnickel *et al.*^37^ – that targets weight bias, applied in a real-world setting. While the initial evaluation presented in Jungnickel *et al.*^37^ only sought to demonstrate the feasibility and general comparability of the mobile app to existing, web-based approaches, the evaluation presented here specifically aims at comparing data obtained from the participants of an obesity-related conference with data recorded and published by Project Implicit over a longer period of time and made available on the Internet.

More specifically, it was of interest whether, similar to the general population, attendees of the conference may, despite their expertise on the subject, still harbor an unconscious bias against individuals with overweight or obesity that they are unwilling to admit.

The following hypotheses were deemed relevant in this context.

#### Hypothesis A

*H*_0_: Within the group of conference participants (G1), there is no difference between the implicit and explicit attitudes.

#### Hypothesis B

*H*_0_: There is no difference in implicit preference across all groups (G1, G2, and G3).

#### Hypothesis C

*H*_0_: There is no difference in explicit preference across all groups (G1, G2, and G3).

## Methods

We compared the endpoints ‘explicit weight bias’ and ‘implicit weight bias’ within and between three populations G1 (attendees at a German obesity conference using the mobile app), G2 (a control group used in a previous technical evaluation – also using the mobile app), and G3 (a subset of the data provided for the German language version of the weight-related IAT by Project Implicit^38^ using a web app). The corresponding variables for explicit attitude and implicit preference were transformed into three expressions (prefer overweight, like both equally/no preference, prefer lean). The evaluation was done solitary and again with respect to dependencies of different dichotomized demographic variables. These included age (up to 34 years, 35 years, and older), gender (female, male), level of education [no higher education, higher education (university level)], BMI category (not overweight, overweight, calculated from self-stated data for body height and weight), and interest in the topic of obesity (not interested, interested, not available for group G3).

### Recruitment and data acquisition

We collected and evaluated data in three independent populations:

1. Group **G1** consisted of attendees of the 36th annual conference of the German Obesity Society (‘Deutsche Adipositas-Gesellschaft’, 8 October 2020 to 10 October 2020 in Leipzig, Germany). This conference primarily hosts professionals active in obesity-related research and care (in both preventive and therapeutic contexts), such as physicians, nurses, nutritionists, and researchers and even administrators, although the conference is also open to laypersons, such as interested patients and non-professional caregivers (e.g. in a family context). Potential participants were randomly approached when passing a booth at the conference fair and asked to perform a mobile, app-based version of the IAT.
2. The second group, **G2**, was composed of data acquired in the technical evaluation phase of the mobile app, using a number of volunteers recruited from the private domain (friends and colleagues). This technical evaluation has been described in detail in Jungnickel *et al.*^37^
3. Data for the third group, **G3**, were prepared from datasets provided by Project Implicit^38^ for those who had participated in a German language, web-based version of the test^39^ between 2006 and 2016.

#### Group 1 (G1): conference attendees

Those interested in participating were informed about the aim of the study, the expected time span necessary for applying the mobile IAT (~10 min), and that the data would be acquired in an anonymous manner without any specific personally identifying items. Those interested were also informed that they could discontinue participation at any time and without consequences.

For administering the mobile IAT, several iPads with the mobile app pre-installed were available. Since the conference was held under pandemic conditions, the devices were thoroughly disinfected by the study staff after each test was performed.

Originally, 93 attendees performed the test. In addition to passing the IAT, the participants were also asked for some demographic data, such as age, weight in kg, height in cm, and their level of education. They were also prompted to indicate whether they personally would prefer those with lean stature to individuals with overweight, or rated both about the same on a 7-point Likert-type scale as used by Project Implicit. Additional data included a rating with respect to the level of interest in the topic of obesity, and whether the participation in the conference was for professional or private reasons because the conference was open to professionals (*n* = 88), and patients and other visitors (*n* = 5) with a nonprofessional background. Although the German Obesity Society was originally founded as a purely medical professional society, the society itself and the annual conference it organizes are now open to a large number of related professions as well.

We therefore refrained from asking the participants to specify their exact profession and field of work in order not to overtax their patience when filling out the survey. Attendees with a non-professional background were excluded from all further evaluations, as were those participants for whom any of the demographic information was missing. Altogether, *n* = 85 datasets for attendees were analyzed. Demographics and the answers to the weight and obesity-related questions were acquired on the same iPads that were used for the IAT.

#### Group 2 (G2): control group

Acquisition of the data for the control group was described previously.^37^ The informed consent process and the app employed corresponded to the approach in the conference group; only the demographic item for professional participation was omitted as it was not applicable for participants in G2. The data were filtered for completeness, and *n* = 51 datasets were available for further evaluation.

#### Group 3 (G3): project implicit data

The subset of data in G3 was compiled from the data provided by the openly accessible dataset and codebook repository^38^ for data acquired by Project Implicit using the web-based version of the IAT in German language^39^ between the years 2006 and 2016.

As there were some inconsistencies with respect to the relevant variables within this dataset, we decided to filter the provided dataset using the following parameters to obtain a plausible subset:

1. Data were only used from 28 April 2008 onward, because before that date, various variable encodings were not fully compatible with the encodings employed later on (and in our own data).
2. Data sets missing any of the relevant demographic variables (i.e. age, gender, education, implicit and explicit ratings) were removed as well.
3. Finally, plausibility checks were applied. Data sets for test participants who had specified to be younger than 18 years of age or older than 80 years were excluded. Participants with implausible values for reported weight (accepted range: 20–300 kg), height (accepted range: 130–220 cm) or BMI (as a ratio of the two values, accepted range: 10–70) were also filtered out.

After filtering, *n* = 13,813 datasets remained for further analysis.

### Tools used in the study

We utilized the concept of the IAT described by Greenwald *et al.*^19,20^ albeit somewhat controversial, this widely used psychological method analyzes unconscious preferences of test subjects by classifying response times needed to perform preference-related tasks. The concept exploits the paradigm that response times increase when participants attempt to consciously modify their responses to certain combinations of concepts and attributes (when these are not consistent with their subconscious attitudes), as opposed to combinations that they subconsciously find more appropriate.

A self-developed, mobile ResearchKit-based^40^ native IAT app for the iOS platform with the Lean–Obese IAT was used for group G1. On a side note, this test is commonly referred to as the Thin–Fat IAT, but to use less stigmatizing vocabulary and to refer to terms commonly used in the community of professionals active in obesity-related contexts, we decided to use ‘lean’ and ‘obese’ to refer to the weight-related strata throughout this article.

The app had been evaluated in a preliminary study (under review, Jungnickel *et al.*^37^), resulting in the dataset for group G2 (control group). In the validation study, we found that the IAT app delivered comparable results with respect to implicit ratings as a survey administered using a web-based version. This web-based version was deployed on a Linux server and was based on both experimental materials provided for the Weight IAT^41^ and example code for a minno.js-based test available at The Docsy Authors.^42^ The web-based version closely followed the current Weight IAT instance localized for the United States. The silhouettes images in use for the self-deployed web-based version corresponded to those used in the US version of the test and were used in the mobile IAT app as well. The positively and negatively connoted terms – eight for each category – used throughout the data acquisition however corresponded to those used in the German Web IAT.

The mobile tests for both G1 and G2 were conducted on Apple iPad devices (eighth generation, 10.2" display) running the latest iPadOS versions that were available at the time of the respective data acquisition.

### Data description

For data description, absolute frequencies and percentages, and means and standard deviation (SD) values are reported.

### Hypotheses testing

For hypothesis testing, Pearson’s *χ*^2^ test^43^ with *α *= 0.05 and *β *= 0.80 was used for nominally scaled values. Cramér’s *V* coefficient as introduced in Cramér^44^ was used as an effect estimator for threefold nominally scaled variables.

### Binary logistic regression

Binary logistic regression was used to analyze which variables had an effect and to what degree, on the relative likelihood of expressing a weight-based preference. For this purpose, the binary-coded variables gender (female, male), age (up to 34 years, 35 years, and older), education [no higher education, higher education (university level)], BMI (not overweight, overweight), and interest in the topic of obesity (not interested, interested) were integrated into the model [inclusion, reference categories: male, 35 years of age and older, higher education (university level), no interest, overweight].

Groups G1 and G2 were combined for the regression analysis because, in addition to the identical survey method, they included the characteristic ‘interest’ that was lacking in the historical control group G3. The correlation analysis was performed with the chi-square test according to Pearson^43^ with *α *= 0.05 and *β *= 0.80, and as an effect parameter for binary nominally scaled variables, the *φ* coefficient Cramér^44^ was used.

### Data evaluation

The available data were evaluated using R 4.1.1^45^ with additional libraries, such as ‘gtsummary’, ‘dplyr’, ‘rcompanion’,^46–48^ and SPSS 26.^49,50^

## Results

We analyzed *n* = 85 full data sets for the conference participants, *n* = 51 for the control group, and *n* = 13,813 for the data provided by Project Implicit (PI) (Table 1).

**Table 1.** Data description for the three groups: G1 – conference, G2 – control group, G3 – data for German participants of Project Implicit, filtered for complete and plausible data.

Characteristic	Conference, *n* = 85	Control group, *n* = 51	Project Implicit (PI), *n* = 13,813
Age			
M (SD)	41.9 (12.9)	34.9 (4.7)	28.6 (10.1)
Age cutoff, *n* (%)			
Up to 34 years	31 (36)	17 (33)	10,769 (78)
35 years and older	54 (64)	34 (67)	3044 (22)
Sex, *n* (%)			
Female	58 (68)	21 (41)	8979 (65)
Male	27 (32)	30 (59)	4834 (35)
Education, *n* (%)			
No higher education	15 (18)	16 (31)	6270 (45)
Higher education (university level)	70 (82)	35 (69)	7543 (55)
BMI category, *n* (%)			
Not overweight	58 (68)	31 (61)	10,231 (74)
Overweight	27 (32)	20 (39)	3582 (26)
Interest in obesity, *n* (%)			
Not interested	11 (13)	48 (94)	0 (NA)
Interested	74 (87)	3 (6)	0 (NA)
PI: data unavailable	0	0	13,813
Explicit attitude, *n* (%)			
Prefer overweight	2 (2)	1 (2)	485 (4)
Like both equally	51 (60)	18 (35)	5091 (37)
Prefer lean	32 (38)	32 (63)	8237 (60)
Implicit preference (IAT), *n* (%)			
Prefer overweight	3 (4)	0 (0)	766 (6)
No preference	7 (8)	5 (10)	1429 (10)
Prefer lean	75 (88)	46 (90)	11,618 (84)

BMI, body mass index; SD, standard deviation.

Participants for both the Conference and Project Implicit groups were predominantly female (conference: *n* = 58 or 68%, Project Implicit: 8979 or 65%), while for the control group, there was a much higher proportion of males who had participated (30 or 59%). With an average of 41.9 ± 12.9 years of age, those who had attended the conference were also older than those in the other two groups (control group: 34.9 ± 4.7 years; Project Implicit data: 28.6 ± 10.1 years). In all three groups, those with a higher education (university level) dominated (G1: 70, 82%; G2: 35, 69%; G3: 7543, 55%) to varying degrees. A quarter of the participants in G3 (3582, 26%) and higher proportions in G1 and G2 fell into the overweight category (G1: 27, 32%; G2: 20, 39%). The smaller proportions of individuals with overweight in G1 and G3 may be explained by the age structure (with participants being much younger in G3) and the professional background of the participants in G1. In addition, the predominance of female participants in G1 and G2 may have played a role with respect to BMI.

### Comparison of implicit and explicit weight bias

Anti-obese biases are apparent in all three groups (Tables 2 and 3). The decisive difference is in the significantly higher percentage (51/85 participants, 60%) of those who reported an explicit attitude of ‘like both equally’ in group G1, compared with groups G2 (35%, 18/51) and G3 (37%, 5091/13813 participants) while there were rather constant percentages toward an implicit rating of ‘prefer lean’ in all three groups (G1: 88%, 75/85; G2: 90%, 46/51; G3: 84%, 11,618/13,813; also see Table 1).

**Table 2.** Cross-tabulation of explicit and implicit (D-score based) attitude category groups for G1, G2, and G3.

		Implicit preference (IAT)			
Group	Characteristic	Prefer overweight	No preference	Prefer lean	Total
G1: conference	Explicit attitude, *n* (%)				
	Prefer overweight	1 (1)	0 (0)	1 (1)	2 (2)
	Like both equally	0 (0)	3 (4)	48 (56)	51 (60)
	Prefer lean	2 (2)	4 (5)	26 (31)	32 (38)
	Total, *n* (%)	3 (4)	7 (8)	75 (88)	85 (100)
G2: control group	Explicit attitude, *n* (%)				
	Prefer overweight	0 (0)	0 (0)	1 (2)	1 (2)
	Like both equally	0 (0)	1 (2)	17 (33)	18 (35)
	Prefer lean	0 (0)	4 (8)	28 (55)	32 (63)
	Total, *n* (%)	0 (0)	5 (10)	46 (90)	51 (100)
G3: Project Implicit	Explicit attitude, *n* (%)				
	Prefer overweight	74 (1)	96 (1)	315 (2)	485 (4)
	Like both equally	373 (3)	667 (5)	4051 (29)	5091 (37)
	Prefer lean	319 (2)	666 (5)	7252 (53)	8237 (60)
	Total, *n* (%)	766 (6)	1429 (10)	11,618 (84)	13,813 (100)

G1: Pearson’s chi-square test: *χ*^2^ = 16.6, *df* = 4, *p* < 0.01, Cramér’s *V* = 0.313.

G2: Pearson’s chi-square test: *χ*^2^ = 0.739, *df* = 2, *p* = 0.691, Cramér’s *V* = 0.12.

G3: Pearson’s chi-square test: *χ*^2^ = 322, *df* = 4, *p* < 0.001, Cramér’s *V* = 0.108.

**Table 3.** Comparison of explicit and implicit preferences between G1, G2, G3, and G1, G2.

Groups	Characteristic	*df*	*χ* ^2^	*p* value	Cramér’s *V*
G1, G2, G3	Explicit attitude	4.00	20	<0.001	0.027
	Implicit preference (IAT)	4.00	4.2	0.4	0.012
G1, G2	Explicit attitude	2.00	8.1	0.017	0.244
	Implicit preference (IAT)	2.00	1.9	0.4	0.118

G1, conference group; G2, control group; G3, Project Implicit data.

### Hypothesis testing

#### Hypothesis A

Regarding weight bias (explicit and implicit), for the conference attendees included in group G1, the explicitly stated attitude does not correspond to the implicit preference; Table 2, left side: *χ*^2^(4) = 16.6, *p *< 0.01, Cramér’s *V* = 0.313. Within G1, the explicit attitude and the implicit preference are different (*H*_0_ declined).

#### Hypothesis B

The implicit preference is not different in all three groups [Table 3, G1, G2, G3, *χ*^2^(4) = 4.2, *p* = 0.4, Cramér’s *V* = 0.012]. *H*_0_ is therefore accepted. Also, there are no notable differences when comparing only the mobile app–based groups [Table 3, G1, G2, *χ*^2^(2) = 1.9, *p* = 0.4, Cramér’s *V* = 0.118].

#### Hypothesis C

The explicit attitude differs in all three groups [Table 3, G1, G2, G3, *χ*^2^(4) = 20, *p *< 0.001, Cramér’s *V* = 0.027]. Hypothesis *H*_0_ is thus declined. Again, this does not change when restricting the comparison to the two app groups [Table 3, G1, G2, *χ*^2^(2) = 8.1, *p* = 0.017, Cramér’s *V* = 0.244).

### Binary logistic regression

The correlation analysis, Table 4, detected a statistically significant correlation for ‘interest in the topic of obesity’, with a medium effect [*χ*^2^(1) = 13, *p *< 0.001, *φ *= 0.3], and a small effect for gender [*χ*^2^(1) = 8.1, *p* = 0.004, *φ* = 0.24]. Descriptively, age, education, and BMI showed correlations with minimal effect sizes. Nevertheless, for the sake of completeness, all of these variables were included in the binary logistic regression.

**Table 4.** Correlations for the conference and control group participants (G1, G2) *versus* explicit attitude, recoded in binary format.

Characteristic	‘Prefer overweight’ or ‘like both equally’, *N* = 72	‘Prefer lean’, *N* = 64	*χ* ^2^	*p* value	*ϕ*
Sex, *n* (%)			8.1	0.004	0.24
Female	50 (69)	29 (45)			
Male	22 (31)	35 (55)			
Age cutoff, *n* (%)			0.04	0.8	0.02
Up to 34 years	26 (36)	22 (34)			
35 years and older	46 (64)	42 (66)			
Education, *n* (%)			0.42	0.5	0.06
No higher education	18 (25)	13 (20)			
Higher education (university level)	54 (75)	51 (80)			
BMI category, *n* (%)			1.1	0.3	0.09
Not overweight	50 (69)	39 (61)			
Overweight	22 (31)	25 (39)			
Interest in obesity, *n* (%)			13	<0.001	0.30
Not interested	21 (29)	38 (59)			
Interested	51 (71)	26 (41)			

BMI, body mass index.

As a result of the logistic regression analysis (Table 5), the feature levels ‘female’ [Exp(B): 0.437, lower CI: 0.202–upper CI: 0.944, *p *= 0.35] and being ‘interested’ in the topic of obesity [Exp(B): 0.284, lower CI: 0.134–upper CI: 0.606, *p *= 0.001] were of significant influence. Based on the odds ratio (OR), compared with men, women were more than two times less likely to favor individuals of lean-to-normal stature over individuals living with obesity (factor 2.3 or 1/0.437). Also, being interested in the topic of obesity reduced the explicit bias against persons with overweight by the factor 3.5 (1/0.284) in comparison with those who were not interested in the topic.

**Table 5.** Result of the binary logistic regression using the dependent variable ‘Explicit attitude’ (recoded in binary format, ‘like both equally’ or ‘prefer overweight’ *versus* ‘prefer lean’) along with the covariates sex, age, education, BMI, and interest in obesity (all recoded as binary). Cox and Snell *R*^2^: 0.136, Nagelkerke’s *R*^2^: 0.181.

Characteristic	Regr. coeff.	Std. error	Wald	*df*	Sig.	Exp(B)	Upper CI	Lower CI
Gender	−0.828	0.393	4.43	1	0.035	0.437	0.202	0.944
Age cutoff	−0.284	0.414	0.472	1	0.492	0.753	0.334	1.69
Education	−0.216	0.425	0.258	1	0.611	0.806	0.35	1.85
BMI	−0.494	0.461	1.15	1	0.284	0.61	0.247	1.51
Interest in obesity	−1.26	0.386	10.6	1	0.001	0.284	0.134	0.606
Constant	1.5	0.587	6.52	1	0.011	4.48		

BMI, body mass index; CI, confidence interval.

There was no significant influence of BMI, age, or education for the analyzed sample (Table 5). The model showed an acceptable fit (Cox and Snell *R*^2^: 0.136, Nagelkerke’s *R*^2^: 0.181).

## Discussion

### Principal findings

Based on the IAT, our results support an implicit anti-obese bias for the conference participants (G1) and for both control groups (G2, G3).

It was interesting that for G1, there was a large proportion of participants who expressed a neutral explicit preference. In our study, 60% of the participants in G1 explicitly stated to ‘like both equally’, while implicit ratings were comparatively stable across all groups, with a predominant preference of lean over overweight individuals (G1: 88%, G2: 90%, G3: 84.11%) and a lesser proportion of neutral ratings (G1: 8%, G2: 10%, G3: 10%; see Table 1). The explicit anti-obese bias in G1 was much less apparent than in G2 and G3, while the implicit anti-obese bias was found to be comparable in all three groups.

With respect to implicit weight bias, the results of those attending the conference (G1), who used our ResearchKit-based implementation of the IAT, were in line with those obtained by other authors for participants from the medical field,^15^ and this is independent of whether web-based,^32,51^ desktop,^52^ paper and pencil-based^29,30,36,53^ or unspecified^54,55^ versions of the IAT were used. In addition, the results shown for G2 are also in line with the general population,^56^ again, independent of the underlying platform.

The almost constant implicit preference over all groups may be interpreted as a result of an early learned and very stable aspect of populations in industrialized nations. Schupp and Renner^57^ were able to confirm the implicit nature of an anti-obese bias based on brain potential recordings. The authors indicated an unconscious and spontaneous anti-obese bias that does not necessarily correspond to explicit opinions, which Schupp and Renner described as ‘inevitable as a reflex’.^57^ There is evidence that this ‘reflex’ may be hard to overcome, even with well-intentioned educational interventions that sometimes fail to reduce obesity-related prejudice.^58^

While the implicit preference seems to be a persistent factor in the population, the explicit attitude inherits much greater plasticity. Both confirmed aspects of our work are in accordance with the literature. The relatively neutral explicit assessments given by the conference attendees (G1) may be related to their professional and keen interest in the obesity topic, which leads to greater understanding toward individuals with overweight or obesity. In our study, we could identify interest in the obesity topic (or lack thereof) as a possibly relevant variable with a 3.5 chance of having an explicit weight bias (lean to overweight) when the participant did not report being interested. Weight, (general) educational level, and age, in contrast, seem not to be relevant.

In the literature, there are also some indications that variable explicit ratings may depend on the affected area of healthcare and/or the rating tool being applied. For example, in a review published by Lawrence *et al.*,^15^ explicit bias was commonly reported for healthcare practitioners using various scales, such as the ‘Fat Phobia Scale’, the ‘Attitudes Toward Obese Persons Scale’, or the ‘Antifat Attitudes Scale’, while there was no such bias for studies assessing nurses aided by the ‘Nurses Toward Overweight and Obese Patients Scale’. For our participants in G1, apart from the simplicity of the single-question assessment (which was used to remain comparable to the data available for G2 and G3), another factor contributing to the large percentage of those with a neutral explicit rating may also have been the setting at a professional conference. More specifically, fear of being observed by peers while giving the answer (however unfounded) may have contributed to participants giving a more socially acceptable answer.

In our data, gender was of less, but still notable influence: males seem to have a 2.3 higher chance of expressing an explicit weight bias than females. For some authors (e.g. Sabin *et al.*^32^, using US-based data), differences between both genders in this regard were less pronounced, while others^59^ found (geographically depended) differences when comparing female and male participants in a multinational setting (the United States, Canada, Iceland, and Australia), with females always exhibiting less bias than men, albeit to a varying degree. Further research with larger populations is needed to confirm these findings.

However, there is the possibility that an unconscious, implicit anti-obese bias could be underestimated by professionally interested individuals. Even those working in health care, who are (or in any case, should be) educated about conditions, such as obesity, being a chronic disease, are not fully immune to the prejudices that prevail in the society they are part of previous studies.^17,29,32,36,52,60,61^ Stereotypes and the often negative characteristics they are associated with may be ingrained from childhood on and may thus be hard to overcome.

### Limitations

#### Recruitment

The number of participants recruited for both G1 and G2 was smaller than we had hoped for during the planning stage. For both groups, the tests were administered during the ongoing coronavirus pandemic, with ensuing consequences, such as many participants attending the conference remotely and thus being unavailable for recruitment at the conference location. We calculated the post hoc powers for hypotheses A, B, and C with G*Power 3.1 and rated these acceptable [Hypothesis A, 1−*β* = 0.77 for *χ*^2^(4) two-tailed test, *α* = 0.05, *n* = 85; Hypotheses B and C, 1−*β* = 0.91 for *χ*^2^(4), two-tailed test, *α* = 0.05, *n* = 130].

#### Representativeness

As we did not have access to the overall demographic data for those visiting the conference, we cannot say with certainty whether the participants were representative for all attendees. Although the study was anonymized, we cannot guarantee potential influences based on the study setting. Nevertheless, we believe that while these aspects may have exerted some influence, we believe that in comparison to the Project Implicit (PI) data, the setting may have improved the acquired demographic data, as the participants were probably more honest due to the chance of being caught with obvious inaccuracies (e.g. related to gender or age, although the study manager did not actually verify these data at the time of acquisition). This is, however, open to debate, as other researchers believe that eliminating the influence of the study staff while filling out a web-based survey may actually reduce related bias and improve data quality compared with one-on-one contact with the study staff.^62–64^

#### Setting

Also, noise and other distractions common at a conference exhibition, such as other attendees walking by, may have influenced the test takers during test performance, which could have had an impact on the measured data and thus the IAT results. Participants also had the chance to discuss the test with other participants or others during administration. However, the chances that participants took part twice were low, due to the face-to-face contact with the study manager.

In addition, while we would have liked to also verify our results with additional tools similar to the ones mentioned by Lawrence *et al.*,^15^ applying different types of tests per individual would have been impractical in our conference setting, where participants commonly only have a short break between the sessions they attend. Such testing should be considered for future work, for example, with participants in a different setting (e.g. clinical or research releated) where it may be easier to recruit subjects by providing time slots that better suit them and where they can then use multiple tools.

#### Comparability of the project implicit data

With respect to the data for the Project Implicit group, due to the anonymity of the survey on the web, it was not certain to what extent the answers given with respect to demographics actually corresponded to the facts or whether the participants might have deliberately provided false information. This might have been relevant for certain characteristics (e.g. age, gender, height, and weight), where participants may have given misleading answers to better conceal their identity or for modesty reasons. Another concern is that this data may also have included results from individuals working in healthcare, and we were unable to filter these out with any certainty, as on the one hand, the encoding was not conducive in this regard and it was also unclear whether all those who had completed the test had correctly answered this. Furthermore, as our study took place in 2020, we would have preferred to use more recent data for G3, but, for the German version of the weight bias-related IAT, there were only datasets leading up to the year 2016.^38^ While there were more recent datasets (e.g. for the United States) that we could have used, for better comparability, we nevertheless decided to keep using the data for Germany, as we felt that using data from a different geographic region would have had a stronger impact than the fact that the data were not completely up to date. Furthermore, due to the relatively large sample we were able to include for G3, despite having to remove obviously implausible datasets, we believe the related effects to be negligible.

#### IAT

For several reasons, the IAT is seen as somewhat controversial in the literature. For example, the popularity and use of the test grew soon after its introduction, even before its foundations, possibilities, and limitations were understood.^65^ There are also those who discuss whether the scores of the IAT actually represent an individual’s unconscious preference against or toward the subject under consideration, or whether the scores rather depend on one’s socialization,^66^ thus reflecting negative beliefs about certain groups as they are conveyed and reinforced by one’s social circle.^67,68^ However, Schaap *et al.*^66^ argue that even considering such factors, the IAT may nevertheless enable researchers to identify differences in socially ingrained habits, skills, and dispositions ascribed to being part of a specific social group. In the context of our study, however, we believe that a distinction between whether the scores are actually based on unconscious opinion or learned from socialization is unimportant here and that it is the effects that count. This may especially be relevant when considering a bias being determined for an overall group, such as healthcare providers hailing from a similar background. If results obtained from IATs for such a group result point to a specific bias, either based on inherent unconscious or learned reactions, it is to be feared that, for example, a negative attitude – no matter how it is motivated – will also come to bear in everyday situations and may possibly have bias decisions. As mentioned at the outset, especially in a care context, this can have a profoundly negative impact on patients. ^17,18^

Furthermore, although appears to be less vulnerable to attempts at faking than is the case for explicit questionnaires,^69^ it is still susceptible to attempt at deception by users who are familiar with its intricacies. By intentionally and carefully adapting response times for specific stimuli, test subjects may easily influence the calculated scores.^70^ There are also those who doubt the IAT’s test–retest reliability across various time intervals, at least for intra-individual testing.^71^ However, when applied to measuring inter-group prejudice, it has been attributed with adequate overall stability in this regard, and Cooley and Payne,^70^ for example, believe that in such group contexts, the IAT may nevertheless provide valuable insights into a group’s implicit attitudes.

Altogether, we believe that, even though possibly lacking sophistication on an individual basis, the test can be a valuable tool for holding up a mirror to a group, so to speak, and illustrating where there might be problems that can be positively influenced by educational measures,^72^ for example.

#### Analysis

We decided to base the encodings for implicit and explicit attitude on the categorizations found in other literature in connection with the IAT. Here, the originally calculated, so-called D-scores (as continuous variables) are artificially assigned to categorical variables. To facilitate the understanding of the results, we additionally decided to further simplify the categorizations and to only retain tendencies in either direction ( ‘prefer overweight’, ‘prefer lean’) or a neutral opinion ( ‘like both equally’ or ‘no preference’).

This transformation may have lead to a more rigorous discrimination of the variables, with a resulting loss of information. Moreover, the statistical tests applied to these newly transformed variables may have led to a biased estimate of the effects (which are already reported to be low). Future work should aim to avoid these transformations and use more sophisticated methods or the original continuously coded scores.

## Conclusion

The results of the presented work show comparable weight biases – implicit and explicit – of professionals working in the field of obesity between attendees of an obesity-related conference using the mobile version (G1) and a historical control group using a web-based version of the IAT (G3). Prejudices among obesity specialists detected by Teachman and Brownell^29^ appear to prevail even 20 years later and after a decade-long discussion about stigmatization of people with obesity. The finding demonstrates the power of implicit preferences and strengthens our approach to construct modern devices for easier studying IAT in the field.

The professionals included in the study, predominantly with a stated interest in the obesity topic, had a much lower level of an explicit anti-obese bias than was apparent in the two control groups. In industrialized nations, implicit preference is relatively stable in its strong tendency of favoring lean individuals, largely independent of other factors. At least this unconscious bias is hard or even impossible to change, and in a healthcare context, may lead to unintended consequences on decisions made by care providers (with possible negative impact for the affected patients). While explicit attitude may be adequately influenced by educational measures and is also positively influenced by (intrinsic) interest in the topic, raising awareness about the potential dissonance between a weaker explicit and stronger implicit bias in healthcare personnel may help improve care: As the implicit preferences are often ingrained from childhood on, and, for professionals, may unconsciously influence decisions made in the care they provide, they should be made aware of this inconsistency to be able to consciously counteract potential effects. More so, if pharmaceutical entities, regulatory authorities, and health insurances are also prone to those prejudices, irrational decisions regarding the development, approval, and payment of obesity-directed therapies, such as weight loss drugs may directly be affected. It would be of interest to prove or disprove any actual effects of raising this awareness on the care process in further studies.

Executing the IAT on a mobile platform with a higher number of participants after the pandemic could confirm our findings. Also comparing the original terms ‘fat’ and ‘thin’ used by Project Implicit, compared to the less stigmatizing vocabulary used in this study ( ‘obese’ and ‘lean’) may be of interest.

From a technical point of view, as the official ResearchKit framework by Apple is only available on iOS-based devices (i.e. iPhones, iPads, and iPod touch devices) and the Apple Watch, we were only able to conduct our research on iPad devices in our study. For IAT-related research targeting a larger audience, which would, for example, be conceivable if a study app were to be distributed through the app stores of not only the iOS but also the Android platform, or in settings where iOS-based devices might be too costly, it would be helpful to implement the test for this second platform as well. As there are similar frameworks available for the Android ecosystem, such as the ResearchStack project^73^ that promises to help developers with porting their ResearchKit-based apps to the Android environment, pose fewer limitations to mobile data acquisition, which may include similar conferences for healthcare professionals.
